# A New TGF-β1 Inhibitor, CTI-82, Antagonizes Epithelial–Mesenchymal Transition through Inhibition of Phospho-SMAD2/3 and Phospho-ERK

**DOI:** 10.3390/biology9070143

**Published:** 2020-06-28

**Authors:** Ji-Hoon Jeong, Hyunhee Kim, Seung-Ho Park, Hayeon Park, Minseok Jeong, Sungmin Kwak, Gi-Jun Sung, Ji-Hye Song, Younghwa Na, Kyung-Chul Choi

**Affiliations:** 1 Department of Biomedical Sciences, Asan Medical Center, AMIST, University of Ulsan College of Medicine, Seoul 05505, Korea; max1431@naver.com (J.-H.J.); ttlok1816@naver.com (H.K.); mylove9322@hanmail.net (S.-H.P.); mintty3@naver.com (H.P.); alstjr9291@naver.com (M.J.); bigawa88@naver.com (S.K.); gjsung89@gmail.com (G.-J.S.); ji.mayfly@gmail.com (J.-H.S.); 2 College of Pharmacy, CHA University, Pocheon 487-010, Korea

**Keywords:** TGF-β1, TGF-β1 inhibitor, EMT, migration, invasion, chalcone

## Abstract

Transforming growth factor-β1 (TGF-β1) is highly expressed in the tumor microenvironment and known to play a multifunctional role in cancer progression. In addition, TGF-β1 promotes metastasis by inducing epithelial–mesenchymal transition (EMT) in a variety of tumors. Thus, inhibition of TGF-β1 is considered an important strategy in the treatment of cancer. In most tumors, TGF-β1 signal transduction exhibits modified or non-functional characteristics, and TGF-β1 inhibitors have various inhibitory effects on cancer cells. Currently, many studies are being conducted to develop TGF-β1 inhibitors from non-toxic natural compounds. We aimed to develop a new TGF-β1 inhibitor to suppress EMT in cancer cells. As a result, improved chalcone-like chain CTI-82 was identified, and its effect was confirmed in vitro. We showed that CTI-82 blocked TGF-β1-induced EMT by inhibiting the cell migration and metastasis of A549 lung cancer cells. In addition, CTI-82 reduced the TGF-β1-induced phosphorylation of SMAD2/3 and inhibited the expression of various EMT markers. Our results suggest that CTI-82 inhibits tumor growth, migration, and metastasis.

## 1. Introduction

Transforming growth factor-β1 (TGF-β1) is a cytokine that plays various functions in tissues. Known as a member of the family of growth and differentiation factors, TGF-β1 regulates diverse cellular functions, such as proliferation, differentiation, and apoptosis [1,2]. TGF-β1 binds to TGF-β receptor II at the cell membrane, and then TGF-β receptor II is complexed with TGF-β receptor I and phosphorylated [3]. The major signal transduction pathway in cells of the TGF-β family is driven by SMAD family proteins. The phosphorylated TGF-β receptor complex phosphorylates SMAD2 and SMAD3, which subsequently form a complex with SMAD4. This complex translocates to the nucleus and acts as a transcription factor. TGF-β1 inhibits proliferation in normal epithelial cells, but this regulation is not observed in cancer cells. In addition, TGF-β1 induces epithelial–mesenchymal transition (EMT) in the epithelium in cancer cells, leading to increased cell motility and invasiveness [4,5,6,7].

EMT is associated with morphological changes and is an important process in cancer cells. The epithelial phenotype is transformed into a mesenchymal fibroblast phenotype that is facilitated by mesenchymal characteristics, including increased migration and invasive abilities [8,9,10]. Since cancer cells undergo numerous extracellular stimulations in vivo, inhibiting TGF-β1-induced EMT-related factors is important in the development of cancer therapy.

EMT plays an important role and is usually associated with cancer tissue metastasis. Epithelial cells that acquire mesenchymal properties exhibit increased mobility and invasiveness, and they promote tumor formation and cancer metastasis [11,12]. It has been found that each phenotype of EMT induced by TGF-β1 is regulated by distinct regulatory factors. The downregulation of E-cadherin promotes mobility and invasion by regulating various transcription inhibitors, such as SNAI1, SNAI2, and ZEB1. During EMT progression, SNAI1 and SNAI2 are overexpressed and inhibit E-cadherin expression [13]. In contrast, mesenchymal markers N-cadherin and fibronectin are not well known and generally not regulated by the above factors [14,15]. In addition, it has been reported that the changes in EMT enhance the secretion of gelatinase matrix metalloproteinase-2 (MMP2) and MMP9 [16,17].

EMT is regulated through various pathways. In particular, the EMT pathway mediated by TGF-β is well known. TGF-β1, which induces EMT, is expressed in most human cells and plays an important role in inhibiting differentiation and growth [18]. Three TGF-β isoforms (TGF-β1, 2, and 3) have been identified that are involved in several important biological phenomena during the processes from onset to progression [19,20]. Since TGF-β1 phosphorylates SMAD2/3/4 complex and induces EMT, TGF-β1 and EMT are highly correlated, and the nuclear translocation of the SMAD complex affects the expression of target genes. Thus, inhibition of SMAD2/3 phosphorylation blocks intracellular signal transduction by TGF-β1, resulting in inhibition of EMT [21,22,23,24].

Reducing the mobility and invasiveness of cells by inhibiting the progression of EMT mediated by TGF-β1 provides a promising strategy to improve the survival rate of patients. There is increasing research to develop TGF-β1 inhibitors using non-toxic natural compounds to suppress TGF-β-induced EMT. Therefore, it is important to develop new inhibitors that can effectively prevent EMT, an essential characteristic of cancer cells.

Chalcone is a plant metabolite commonly found in vegetables, spices, tea, and fruits. It exhibits various biological and pharmacological activities, such as anticancer, anti-inflammatory, anti-infective, immunomodulatory, antibacterial, and antioxidant activates [25]. Natural chalcone compounds have been reported to inhibit cytotoxicity and in vitro cytotoxic activity in prostate cancer [26]. Furthermore, chalcone compounds are potential anticancer agents and inhibitors of cathepsin B and L [27]. Cathepsin represents a group of proteases and is involved in determining the metastasis of cancer cells [28]. In particular, it has been reported that suppression of cathepsin L inhibits EMT induced by TGF-β1 and suppresses cancer mobility and invasiveness [26,29]. We studied the TGF-β1 signaling pathway by screening 82 compounds by further designing and synthesizing chalcone compounds. We aimed to develop inhibitors that inhibit EMT in lung cancer cells through chalcone analogs. As a result, we demonstrated the inhibitory role of CTI-82 during TGF-β1-mediated EMT process in A549 lung cancer cells.

## 2. Materials and Methods

### 2.1. Cells and Reagents

HaCaT cells were cultured in DMEM (Corning, Buffalo, NY, USA) containing 10% fetal bovine serum (FBS; Corning) and 1% antibiotics (Hyclone, Waltham, MA, USA). Human lung cancer A549 cells were cultured in RPMI (Corning) containing 10% FBS and 1% antibiotics, and all cells were cultured in a 37 °C incubator with 5% CO_2_. TGF-β1 was obtained from PeproTech (Rocky Hill, NJ, USA). SB431542 was purchased from Selleckchem (Houston, TX, USA). The following antibodies were used: anti-SMAD2 (5339S), anti-SMAD3 (9523S), anti-p-SMAD2 (S465/467; 3108S), p-SMAD3 (S423/425; 9520S), anti-ERK1/2 (9102S), anti-p-ERK1/2 (T202/Y204; 9101S), anti-p-p38 (T180/Y182; 4631S) (Cell Signaling, Danvers, MA, USA), anti-JNK1/2/3 (ab208035), anti-p-JNK1/2/3 (Y185/Y185/Y223; ab76572), anti-p38 (ab32142) (abcam, Cambridge, MA, USA), anti-N-cadherin (sc-59987), anti-E-cadherin (sc-71009), anti-SNAI2 (sc-166476), anti-MMP9 (sc-13520), anti-COL1A2 (sc-393573), anti-PAI-1 (sc-5297) (Santa Cruz Biotechnology, Dallas, TX, USA), and anti-β-actin (Sigma-Aldrich, St. Louis, MO, USA). All secondary antibodies were purchased from Thermo Scientific (Waltham, MA, USA). TransIT-LT1 transfection reagent was purchased from Mirus Bio (Madison, WI, USA).

### 2.2. Cell Viability Assays

To determine the cytotoxicity of CTI-82 in A549 cells, cell viability was measured by one of the conventional measurement methods, the MTT reduction assay. Briefly, A549 cells were seeded at a density of 1 × 10^3^ cells/well in a 96-well plate. After 24 h of incubation, the cells were co-treated with TGF-β1 and CTI-82 (0, 10, 20, 30, 40, and 50 μM) for 48 h. Cells were treated with 15 mL of MTT solution (2 mg/mL) for 60 min at 37 °C, and the absorbance at 570 nm was measured and recorded on a microplate reader (Model 550, BIO-RAD Laboratories, Hercules, CA, USA). All MTT assay results were expressed as the mean (±SD) of three independent experiments.

### 2.3. Reporter Gene Assay

A549 cells were transiently transfected with the reporter plasmid pSBE4-Luc to determine the transcriptional activity of SMAD. The β-gal luciferase reporter plasmid was co-transfected as an internal control. Then, the cells were harvested, and luciferase activity was measured according to the manufacturer’s instructions (Promega, Madison, WI, USA). All reporter activity was normalized to β-gal luciferase activity and presented as the mean (±SD) of three independent experiments.

### 2.4. RNA Extraction and Quantitative Real-Time PCR

Total RNA was isolated using an RNA EasySpin kit (Intron Biotechnology, Seongnam-si, Gyeonggi-do, Republic of Korea) according to the manufacturer’s instructions. cDNA synthesis was performed using a cDNA synthesis kit (primescript^®^ RTreagent kit, Takara, Kusatsu, Shiga, Japan) to convert total RNA into cDNA. For real-time PCR, the primers for various genes were designed in Primer-BLAST (NCBI, Bethesda, MD, USA). The primers used were as follows: *E-cadherin* (5′-TGCCCAGAAAATGAAAAAGG-3′, 5′-GTGTATGTGGCAATGCGTTC-3′), *N-cadherin* (5′-CCATCACTCGGCTTAATGGT-3′, 5′-GATGATGATGCAGAGCAGGA-3′), *MMP2* (5′-ACATCAAGGGCATTCAGGAG-3′, 5′-GCCTCGTATACCGCATCAAT-3′), *MMP9* (5′-CATCGTCATCCAGTTTGGTG-3′, 5′-TCGAAGATGAAGGGGAAGTG-3′), *COL1A1* (5′-AGCCAGCAGATCGAGAACAT-3′, 5′-TCTTGTCCTTGGGGTTCTTG-3′), *ZEB1* (5′-TGCACTGAGTGTGGAAAAGC-3′, 5′-TGGTGATGCTGAAAGAGACG-3′), and *GAPDH* (5′-CGCGGGGCTCTCCAGAACATCATCC-3′, 5′-CTCCGACGCCTGCTTCACCACCTTCTT-3′). Real-time PCR was performed using a real-time PCR kit (EBT-1801; HiPi Real-Time PCR 2x Master Mix, ELPIS Bio, Daedeok-gu, Daejeon, Republic of Korea), and all samples were normalized using the ΔΔCt method. In addition, numerical values for all expression levels were expressed as fold changes. All reactions were repeated three times, and relative expression levels and SDs were calculated using Microsoft Excel (Office 365).

### 2.5. Western Blot Analysis

Briefly, after 48 h of treatment with TGF-β1 and CTI-82, A549 and HaCaT cells were washed with PBS, scraped, and harvested. Cell extracts were lysed on ice for 30 min with lysis buffer (10 mmol/L NaF, protease inhibitors, 10 mmol/L sodium pyrophosphate, 150 mmol/L NaCl, 1% NP-40, and 50 mmol/L Tris-HCl (pH 7.5)). After cell lysis, cells were centrifuged at 13,000 rpm for 20 min at 4 °C. The supernatant was transferred to a new tube, and the protein concentration of the whole cell lysate was measured with a Pierce 660 nm protein assay reagent (Thermo Scientific). The samples were separated on 8–12% SDS-PAGE gels and transferred to nitrocellulose membranes. The membranes were blocked by incubation for 2 h with *w/v* non-fat DifcoTM skim milk (BD Biosciences, Franklin Lakes, NJ, USA) in blocking buffer with 1X PBST. The blocked membrane was then incubated with the indicated primary antibodies for overnight at 4 °C. After washing three times with 1× PBST for 10 min each, the secondary antibody was incubated for 1.5 h. After washing three times in the same 1X PBST for 10 min each, protein bands were visualized by the developer. The signal was quantified by ImageJ (Java 1.8.0_112, NIH, Bethesda, MD, USA), and the level of protein expression was normalized to β-actin.

### 2.6. Matrigel Invasion Assay and Wound-Healing Assay

In the Biocoat Martigel invasion chamber (SPL Life Science, Pocheon-si, Gyeonggi-do, Republic of Korea), a Matrigel invasion assay was used to confirm the ability of cells to migrate through the extracellular matrix. A549 cells (2 × 10^4^) were seeded in each well. The cells were then cultured for 12 h prior to co-treatment with CTI-82 and TGF-β1. After incubation for 48 h, non-invaded cells were removed with a cotton swab. The invaded cells were fixed with 100% methanol and stained with 1% crystal violet (Sigma-Aldrich). After staining, the number of invaded cells was counted with a microscope (40×, three random fields per well). Data were expressed as the mean (±SD) of at least three independent experiments.

In the wound-healing assay, A549 cells were cultured to 80% confluency, and then cells were scratched using a 20 µL pipette tip. The scraped cells were removed with DPBS, the media was changed, TGF-β1 and CTI-82 were added, and cells were cultured for 48 h. Wound healing was observed within 48 h of the scratched wounds. The TScratch program (TScratch 1.0) was used to quantify migration by measuring cell surface area. The data were expressed as the mean (±SD) of at least three independent experiments.

### 2.7. Confocal Microscopy

A549 cells were seeded 3 × 10^4^ in a confocal dish (SPL, 101350). The grown A549 cells were fixed in 4% paraformaldehyde for 10 min, permeabilized in 0.3% Triton X-100 for 5 min and blocked with 10% goat serum albumin for 1 h at room temperature. Cells were incubated with the indicated primary antibody at room temperature for 3 h or overnight at 4 °C, washed three times with PBS, and then the secondary antibody was incubated at room temperature for 1 h. Antibodies for immunofluorescence were anti-N-cadherin, anti-E-cadherin, and goat anti-rabbit IgG-FITC (sc-2012, Santa Cruz). DAPI (D5942; Sigma-Aldrich) was used to stain cell nuclei. Imaging of stationary samples was performed in the ZOE Fluorescent Cell Imager (Bio-Rad).

### 2.8. Statistical Analysis

Statistical significance was determined via two-tailed unpaired Student’s *t*-test or one-way ANOVA using Microsoft Excel (Office 365) or Prism (Prism 8.0.1, GraphPad, San Diego, CA, USA) software. A two-tailed *t*-test was used for comparisons between two groups. All data are presented as mean ± standard error. Values were reported as the mean ± SD. *p*-values < 0.05 were considered significant. Detail values are shown in the figure legends.

## 3. Results

### 3.1. Screening of a New TGF-β1 Inhibitor in Chalcone Derivatives

**Figure 1:**
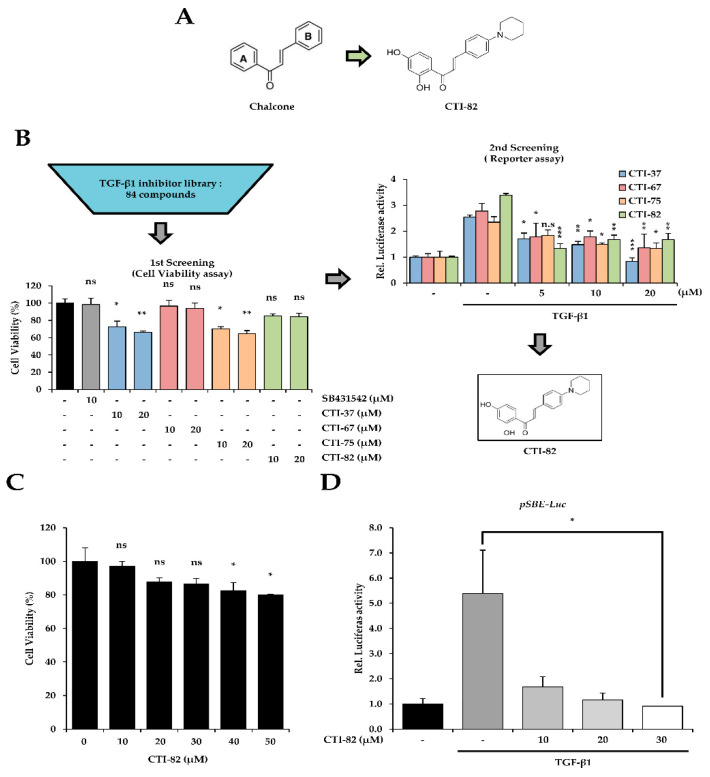
Profiles of the transforming growth factor-β1 (TGF-β1) inhibitor screen. (**A**) Structure of CTI-82 with a chalcone-like backbone as a TGF-β1 inhibitor. (**B**) Flowchart of the screen. CTI-82 from the library was screened by cell viability and reporter assays. The screen was performed as described. (**C**) The dose-dependent effect of CTI-82 treatment for 48 h on the cell viability of A549 cells. (**D**) CTI-82 inhibits the transcriptional activity of SMAD induced by TGF-β1. Cells were treated with TGF-β1 (5 ng/mL) for 2 h, treated with CTI-82, and then cultured for 48 h. SMAD transcriptional activity was analyzed using the pSBE-Luc reporter gene. *** *p*-value < 0.001, ** *p*-value < 0.01, * *p*-value < 0.05, TGF-β1 alone versus the TGF-β1- and CTI-82-treated group for their respective dose points. ns; not significant. The data are expressed as the mean ± SD for triplicates.

Our study investigated the mechanisms and effects of CTI-82, which strongly inhibits EMT induced by TGF-β1 in the human lung cancer cell line A549. Our study demonstrated that CTI-82 inhibits TGF-β1-induced migration and invasion in A549 cells. CTI-82 inhibits TGF-β1-induced SMAD2/3 phosphorylation in A549 cells and HaCaT cells (keratinocytes) and inhibits migration and invasion by reducing EMT. Thus, we suggest that CTI-82 is effective as a new TGF-β1 inhibitor in human lung cancer cells and keratinocytes (Figure 1A). We screened 84 potential TGF-β1 inhibitor candidate libraries designed as chalcone structures. The first screening measured cell viability. When measured by a dose-dependent method, concentrations that resulted in at least 80% cell viability were selected for 48 h. The secondary screening was performed with a luciferase assay. pSBE4-Luc and β-gal plasmids were transfected into A549 cells for 24 h. TGF-β1 was co-treated at a concentration that resulted in 80% or more cell viability selected in the first screening (Figure 1B). As a result of the first screening, cells treated with 10, 20, 30, 40, and 50 μM of CTI-82 for 48 h showed the cell viability of 80% or more. However, at 40 μM, the toxicity significantly appeared at 40 μM (Figure 1C). Therefore, based on these results, the next experiments were performed at a concentration of 30 μM. As a result, when TGF-β1 was treated with CTI-82 in A549 cells for 48 h, the transcriptional activity of SMAD was significantly reduced (Figure 1D). These results suggest that CTI-82 inhibits TGF-β1 signaling by reducing the transcriptional activity of SMAD in the range of up to 30 μM.

### 3.2. CTI-82 Inhibits the Phosphorylation of SMAD2/3 Induced by TGF-β1

**Figure 2:**
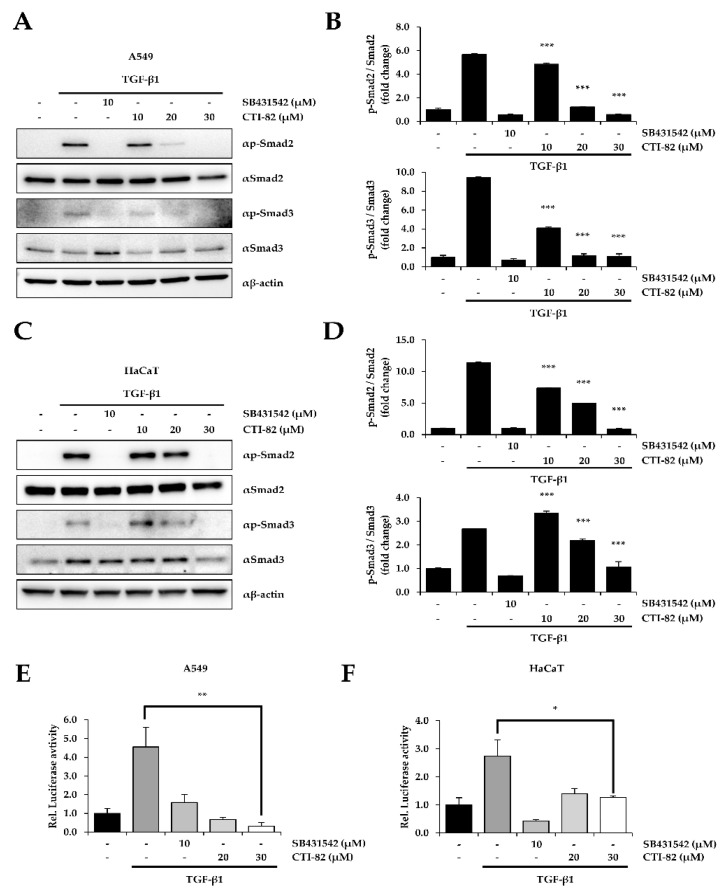
CTI-82 inhibits TGF-β1-induced SMAD2/3 activation. (**A**,**B**) A549 and (**C**,**D**) HaCaT cells were treated with TGF-β1 (5 ng/mL) for 2 h, followed by the indicated concentrations of CTI-82 for 48 h. Proteins in A549 and HaCaT cells were detected by Western blot analysis (left) and densitometric quantification (right) using anti-SMAD2, SMAD3, anti-phospho-SMAD2, and SMAD3 antibodies. (**E**,**F**) CTI-82 effectively inhibits the transcriptional activity of SMAD induced by TGF-β1 (5 ng/mL). After stimulating A549 and HaCaT cells with TGF-β1 for 2 h, cells were treated with different concentrations of CTI-82 for 48 h. The transcriptional activity of SMAD was analyzed using the pSBE-Luc reporter gene. *** *p*-value < 0.001, ** *p*-value < 0.01, * *p*-value < 0.05, TGF-β1 alone versus the TGF-β1- and CTI-82-treated group for their respective dose points. The data are expressed as the mean ± SD for triplicates. (The uncropped blots and molecular weight markers are shown in Supplementary Figure S1).

We confirmed the inhibitory effect of CTI-82 during TGF-β-induced EMT in HaCaT cells, one of the cell lines suitable for studying TGF-β-induced EMT similar to A549 cells. HaCaT cells along with A549 cells are one of the most used cells to study EMT by the various growth factors containing TGF-β1 [30,31]. As a result, we found that CTI-82 significantly inhibited the phosphorylation of SMAD2/3 at selected concentrations in the human lung cancer A549 cells and the human keratinocyte HaCaT cells. After TGF-β1 treatment in A549 and HaCaT cells, CTI-82 was dose-dependently added. SB431542, a TGF-β1 inhibitor, was used as a control. The results showed that in A549 and HaCaT cells treated with TGF-β1 and CTI-82, the phosphorylation of SMAD2/3 was reduced in a concentration-dependent manner (Figure 2A,B). In addition, phosphorylation was reduced to the similar level as SB431542 at 30 μM, and the same result was obtained in HaCaT cells (Figure 2C,D). The luciferase assay using pSBE4-Luc resulted in a dose-dependent reduction in SMAD transcriptional activity induced by TGF-β1 in A549 and HaCaT cells (Figure 2E,F).

### 3.3. CTI-82 Antagonizes TGF-β1-Induced EMT

**Figure 3:**
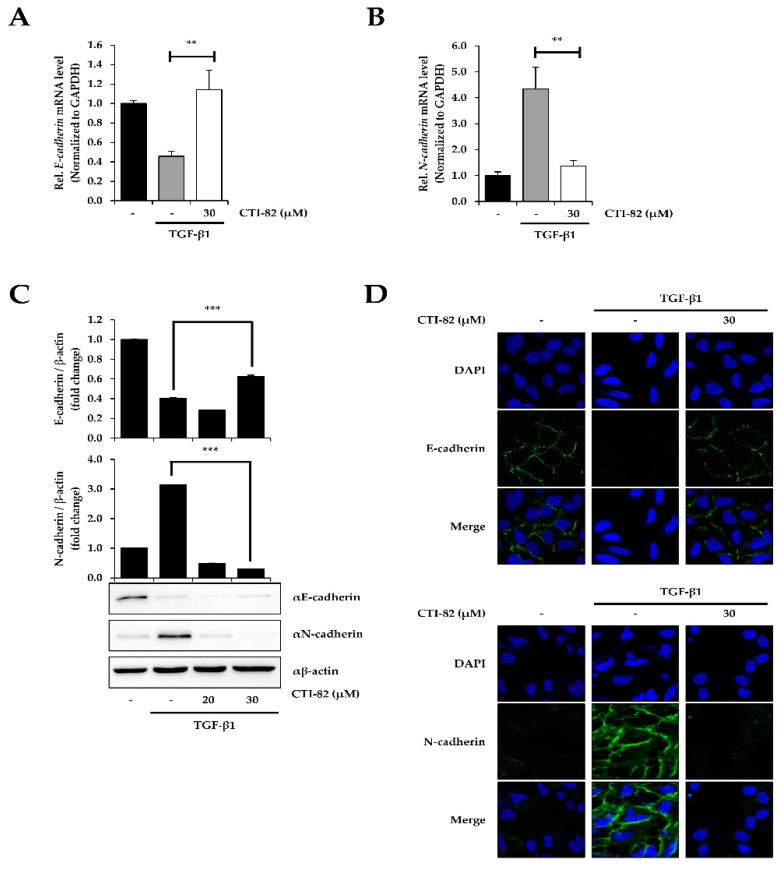
CTI-82 antagonizes TGF-β1-induced epithelial–mesenchymal transition (EMT). (**A**) E-cadherin mRNA expression and (**B**) N-cadherin mRNA expression. A549 cells were treated with TGF-β1 (5 ng/mL) for 2 h, followed by CTI-82 for 48 h. Using quantitative RT-PCR analysis, each gene expression level was analyzed and compared with the control group treated with TGF-β1 alone. (**C**) Proteins in A549 cells were detected by Western blot analysis using E-cadherin and N-cadherin antibodies, and measured by densitometric quantification. (**D**) In A549 cells induced by TGF-β1, CTI-82 increased the expression of E-cadherin and decreased the expression of N-cadherin. The expression levels of E-cadherin and N-cadherin were detected by immunofluorescence analysis using each primary antibody. Nuclear staining was indicated by DAPI. *** *p*-value < 0.001, ** *p*-value < 0.01 versus TGF-β1 alone. The data are expressed as the mean ± SD for triplicates. (The uncropped blots and molecular weight markers are shown in Supplementary Figure S1).

TGF-β1 reduces E-cadherin expression and significantly increases N-cadherin expression [32,33]. In the human lung cancer cell A549, CTI-82 has been shown to inhibit EMT induced by TGF-β1. First, the expression of E-cadherin and N-cadherin was confirmed by quantitative real-time polymerase chain reaction (qPCR). As a result, E-cadherin decreased, and N-cadherin increased significantly in A549 cells stimulated with TGF-β1 alone. On the other hand, when cells were co-treated with CTI-82 and TGF-β1, the level of E-cadherin was restored (Figure 3A), and N-cadherin decreased (Figure 3B). The same results were obtained at 30 μM when E-cadherin and N-cadherin expression was measured via Western blot and densitometric quantification (Figure 3C). The expression of E-cadherin and N-cadherin in A549 cells was confirmed by immunofluorescence microscopy using a confocal microscope. E-cadherin expression was significantly reduced in cells treated with TGF-β1, and normal E-cadherin expression was recovered when cells were co-treated with CTI-82 (Figure 3D). In contrast, N-cadherin was strongly expressed when cells were treated with TGF-β1, but its expression decreased when CTI-82 was added together with TGF-β1. These results demonstrate that CTI-82 strongly increases and decreases E-cadherin expression and N-cadherin expression during TGF-β-induced EMT in the human lung cancer cell A549.

### 3.4. CTI-82 Attenuates the Phosphorylation of ERK1/2 Induced by TGF-β1

**Figure 4:**
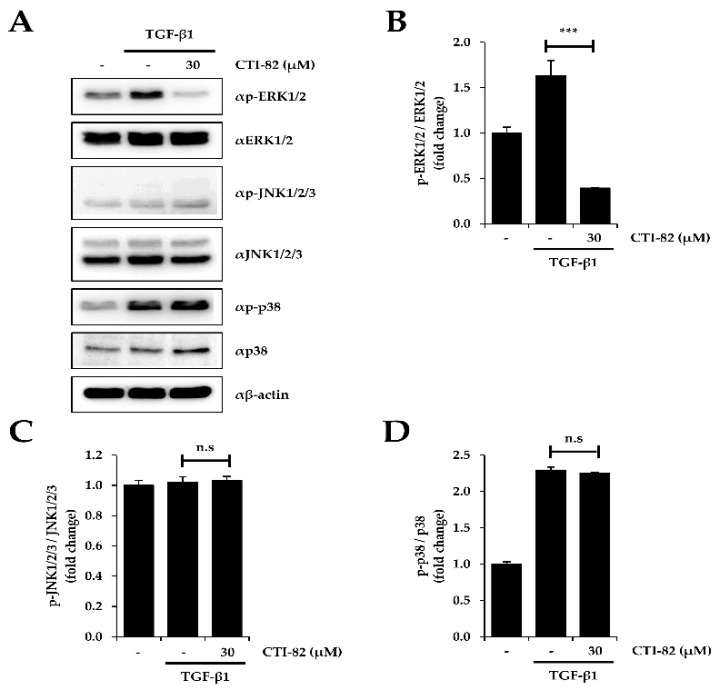
Effect of CTI-82 on mitogen-activated protein kinase (MAPK) signaling pathway in TGF-β1-treated A549 cells. (**A**) Protein expression of p-ERK1/2, p-JNK1/2/3 and p-p38. CTI-82 attenuates MAPK signaling induced by TGF-β1. A549 cells were treated with TGF-β1 for 2 h, followed by treatment with CTI-82 for 48 h. Western blot analysis was performed with cell lysates. The graphs show densitometric quantification of p-ERK1/2 (**B**), p-JNK1/2/3 (**C**), and p-p38 (**D**). *** *p*-value < 0.001 versus TGF-β1 alone. ns; not significant. The data are expressed as the mean ± SD for triplicates. (The uncropped blots and molecular weight markers are shown in Supplementary Figure S1).

Besides the canonical signaling, TGF-β1 also activates mitogen-activated protein kinase (MAPK) signaling, also known as non-canonical signaling [34]. We confirmed that after treatment with TGF-β1, CTI-82 attenuated the increased phosphorylation of extracellular signal-regulated kinase 1/2 (ERK1/2) in A549 cells. On the other hand, phosphorylation of c-Jun N-terminal kinase 1/2/3 (JNK1/2/3) and p38 were not affected (Figure 4A–D). Collectively, these results suggested that CTI-82 significantly inhibits SMAD and MAPK signaling induced by TGF-β1.

### 3.5. CTI-82 Represses TGF-β1-Induced EMT Transcripts

**Figure 5:**
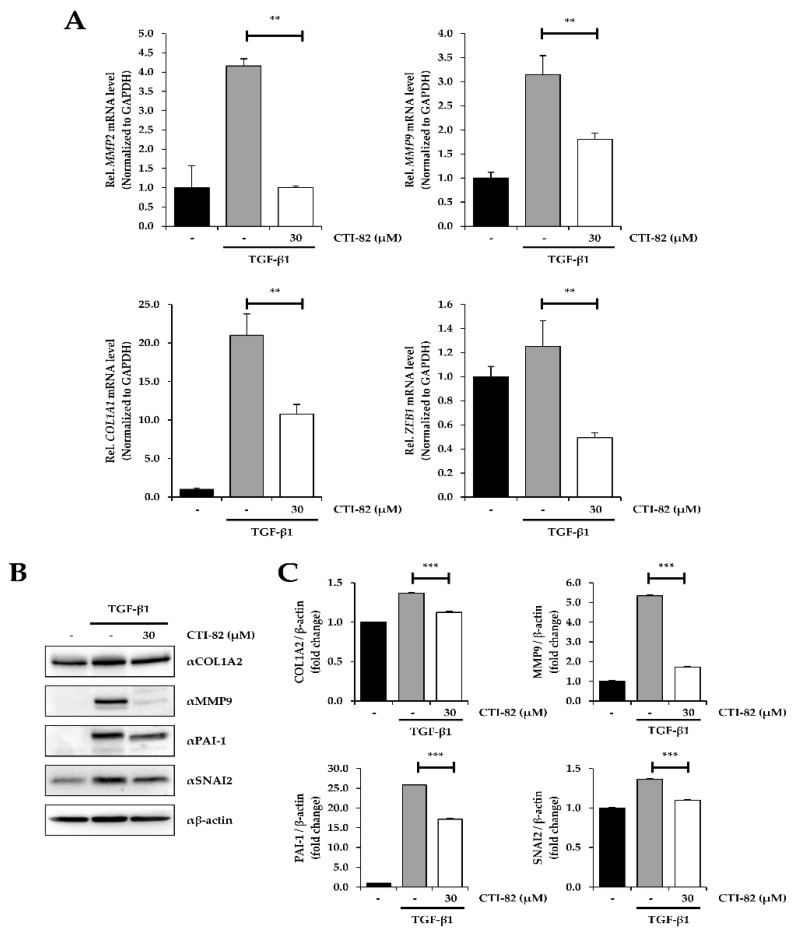
Effect of CTI-82 on TGF-β1-induced EMT-related gene expression. (**A**) Expression of matrix metalloproteinase-2 (MMP2), MMP9, COL1A1, and ZEB1 mRNA. CTI-82 regulates the expression of EMT-related genes induced by TGF-β1. A549 cells were treated with TGF-β1 (5 ng/mL) alone for 2 h, followed by treatment with CTI-82 for 48 h. Using qPCR analysis, the expression level of the genes was analyzed and compared to the control group treated with TGF-β1 alone. (**B**,**C**) Western blot analysis and densitometric quantification of E-cadherin, N-cadherin, COL1A2, MMP9, PAI-1, and SNAI2 in A549 cells. A549 cells were treated with TGF-β1 for 2 h, followed by treatment with CTI-82 for 48 h. Western blot analysis was performed with cell lysates. *** *p*-value < 0.001, ** *p*-value < 0.01 versus TGF-β1 alone. The data are expressed as the mean ± SD for triplicates. (The uncropped blots and molecular weight markers are shown in Supplementary Figure S1).

Expression of various EMT-related genes was confirmed by qPCR and Western blot to verify that CTI-82 blocked EMT induced by TGF-β1. CTI-82 at 30 μM was co-treated with TGF-β1 in A549 cells for 48 h. The qPCR results showed that there were several changes in the expression of EMT markers. Specifically, MMP2, MMP9, COL1A1, and ZEB1 were significantly increased in A549 cells treated with TGF-β1 alone but significantly decreased in A549 cells treated with CTI-82 (Figure 5A). CTI-82 significantly inhibited the expression of various EMT marker proteins, such as COL1A2, PAI-1, and SNAI2 (Figure 5B,C). Taken together, these results demonstrate that CTI-82 strongly inhibits TGF-β1-induced EMT.

### 3.6. CTI-82 Inhibits TGF-β1-Induced Cell Migration and Invasion

**Figure 6:**
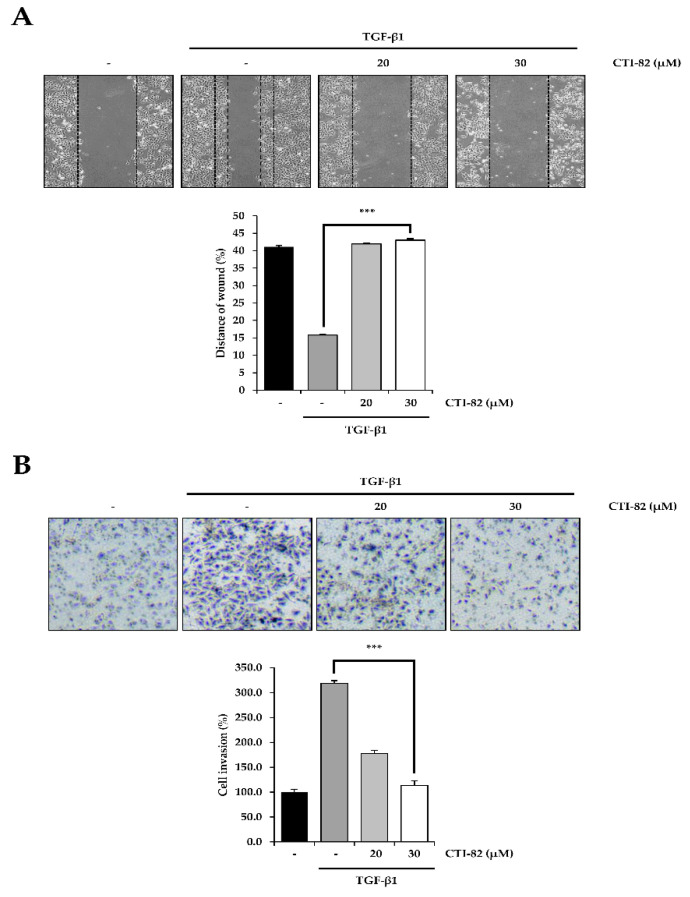
CTI-82 suppresses TGF-β1-induced cell migration and invasion. (**A**) CTI-82 inhibits migration induced by TGF-β1. Wound-healing assay results show the amount of movement in A549 cells treated with TGF-β1 for 2 h and then CTI-82 for 48 h. Quantitative histograms show the average level observed in three random fields for each condition. (**B**) CTI-82 inhibits invasion induced by TGF-β1. A549 cells were treated with TGF-β1 (5 ng/mL) for 2 h, followed by CTI-82 for 48 h. A549 cells invaded after 48 h were detected and analyzed by the Matrigel invasion assay. Invasiveness was measured by counting cells in the matrix layer of the invasive chamber. *** *p*-value < 0.001 versus TGF-β1 alone. The data are expressed as the mean ± SD for triplicates.

Finally, we confirmed that CTI-82 inhibited TGF-β1-induced migration and invasion. First, a cell migration assay was performed. A549 cells were cultured on a 6-well plate until the confluency reached 90% or more. We then scratched the cells with a pipette tip to generate a wound area. After changing the media, CTI-82 was treated with TGF-β1, and cells were cultured for 48 h. Compared with A549 cells treated with TGF-β1 alone, the migration of A549 cells was significantly reduced in the presence of 30 μM of CTI-82 (Figure 6A). In addition, a transwell chamber invasion assay was performed to determine whether CTI-82 inhibited invasion. The insert chamber was coated with Matrigel. A549 cells treated with TGF-β1 alone and A549 cells treated with CTI-82 were incubated for 48 h in uncoated inserts. The cells were then immobilized to measure the invaded cells. A549 cells treated with TGF-β1 invaded the transwell chambers. In contrast, A549 cells treated with CTI-82 exhibited significantly reduced invasion in a dose-dependent manner (Figure 6B).

## 4. Discussion

Natural anticancer compounds inhibit tumor growth and progression by reducing cell migration, invasion, and metastasis [35,36,37,38,39,40,41,42]. Interestingly, chalcone-like chain CTI-82 exhibits several pharmacological effects. It is also used as an anticancer and anti-inflammatory agent and is known as a promising new drug candidate compound with biological activity and stability [27,28,29,43]. Our study showed that CTI-82, a chalcone analog, inhibited the mobility and invasiveness of A549 cells stimulated by TGF-β1.

We first performed a primary screening of a library of 84 potential TGF-β1 inhibitors to determine the concentration range of CTI-82, a chalcone analog. The secondary screening was confirmed by whether the transcriptional activity of SMAD at the selected concentration was inhibited. CTI-82 did not cause the cell cytotoxicity at 30 μM but significantly decreased the transcriptional activity of SMAD in a dose-dependent manner. In the canonical TGF-β signaling pathway, TGF-β1-induced EMT is generally initiated by phosphorylated SMAD2 and SMAD3, which translocate to the nucleus to induce the expression of EMT-related genes. We found that CTI-82, a chalcone analog, inhibited the phosphorylation of SMAD2/3 induced by TGF-β1. The expression of SMAD2/3 phosphorylation was reduced in a dose-dependent manner in A549 cells treated with CTI-82. In addition, both SMAD2/3 expression and transcriptional activity were reduced to levels similar to those in cells treated with the TGF-β1 inhibitor SB431542. Thus, these results indicate that CTI-82 antagonizes the phosphorylation of SMAD2/3 induced by TGF-β1 in the canonical TGF-β pathway.

Additionally, in the canonical signaling pathway, TGF-β1 activates the p38/JNK MAPK signaling, which proceeds through activation of TAK1 by TRAF6. Shr is mobilized by TGF-β receptor I (TGFβR1) to activate the ERK MAPK pathway. Furthermore, TGF-β1 activates phosphatidylinositol-3-kinase (PI3K)/AKT axis [44,45,46,47,48]. Thus, to confirm CTI-82 suppresses the non-canonical signaling pathway, we confirmed that CTI-82 inhibits phosphorylation of non-canonical signaling in addition to canonical signaling pathway. As shown in Figure 4, CTI-82 can partially inhibit not only canonical signaling but also non-canonical signaling by significantly inhibiting phosphorylation of ERK caused by TGF-β1. Thus, we strongly suggest that CTI-82 may effectively inhibit the cancer metastasis and tumorigenesis to EMT process of the growth factor-induced cancer cells.

SMAD2/3 is complexed with SMAD4 and translocated into the nucleus. This complex increases the extracellular matrix (ECM), EMT, mobility, and invasiveness by directly binding to the target gene or inducing associated gene markers such as collagenase, matrilysin, urokinase, and MMPs along with other transcription factors [14,49,50,51,52]. Transcription factors such as SNAI2 and ZEB1 play a very important role in the process of EMT and tumor formation through inhibition of E-cadherin expression [53,54]. In addition to these transcription factors, since TGF-β1 upregulates collagen type I and MMPs in ECM synthesis, this study confirmed that genes involved in the increase in TGF-β1-induced EMT are inhibited [55,56,57,58]. As shown in Figure 5, CTI-82 significantly inhibited the mRNA and protein levels of the transcription factor, collagen, and MMP genes that increase EMT. CTI-82 may be a novel inhibitor of EMT induced by TGF-β1 because it inhibits the mRNA and protein levels of various EMT markers. However, the detailed mechanism by which CTI-82 modulates the TGF-β receptor is unclear. In this study, SB431542, an inhibitor that specifically inhibits TGFβR1 phosphorylation, showed similar effects to CTI-82. Therefore, to inhibit the TGF-β signaling pathway, the TGF-β receptor can be a target of CTI-82.

## 5. Conclusions

In summary, our study demonstrates that CTI-82, a chalcone analog, inhibits EMT by inhibiting the phosphorylation of SMAD2/3 in the canonical TGF-β pathway. In addition, CTI-82 inhibits EMT induced by TGF-β1, suggesting that it may be a novel therapeutic substance to prevent the migration and invasion of lung cancer and keratinocytes.

## Supplementary Materials

The following are available online at https://www.mdpi.com/2079-7737/9/7/143/s1, Figure S1: The whole western blot of Figure 2A,C, Figure 3C, Figure 4A and Figure 5B. The band of western blot of all figures was quantified by densitometry readings.
