# Supplementary material for: A New TGF-β1 Inhibitor, CTI-82, Antagonizes Epithelial–Mesenchymal Transition through Inhibition of Phospho-SMAD2/3 and Phospho-ERK
Source: Biology (Basel). 2020 Jun 28;9(7):143. doi: 10.3390/biology9070143 (PMC7408591; doi:10.3390/biology9070143)
Supplement: Supplementary file 1 [file biology-09-00143-s001.pdf]

Supplementary Materials:

A New TGF-β1 Inhibitor, CTI-82, Antagonizes Epithelial–Mesenchymal Transition through Inhibition of Phospho-SMAD2/3 and Phospho-ERK

Supplementary Figure S1. The whole western blot of Figure 2A, 2C, 3C, 4A and 5B. The band of western blot of all figures was quantified by densitometry readings.

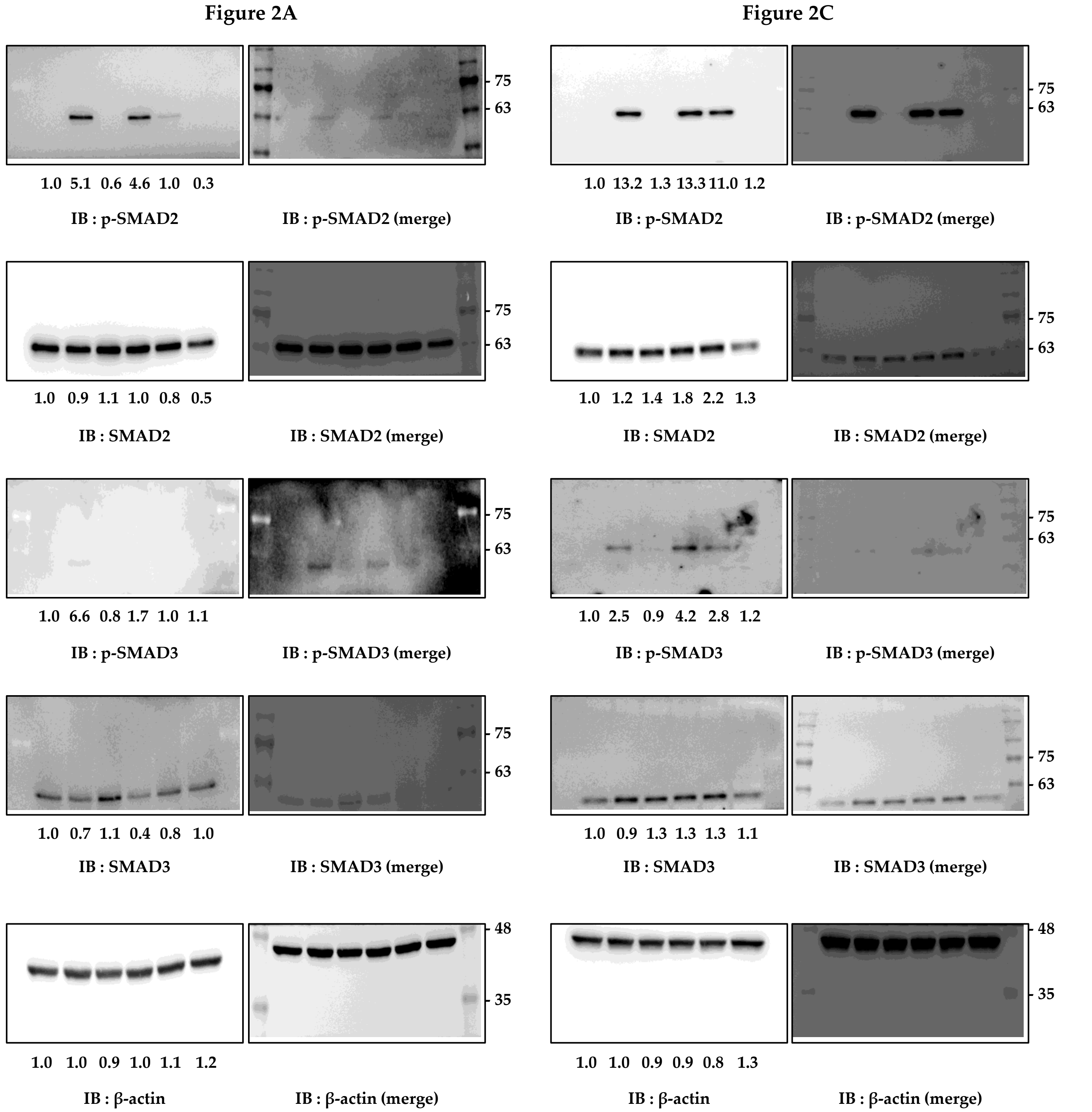

Figure 3C

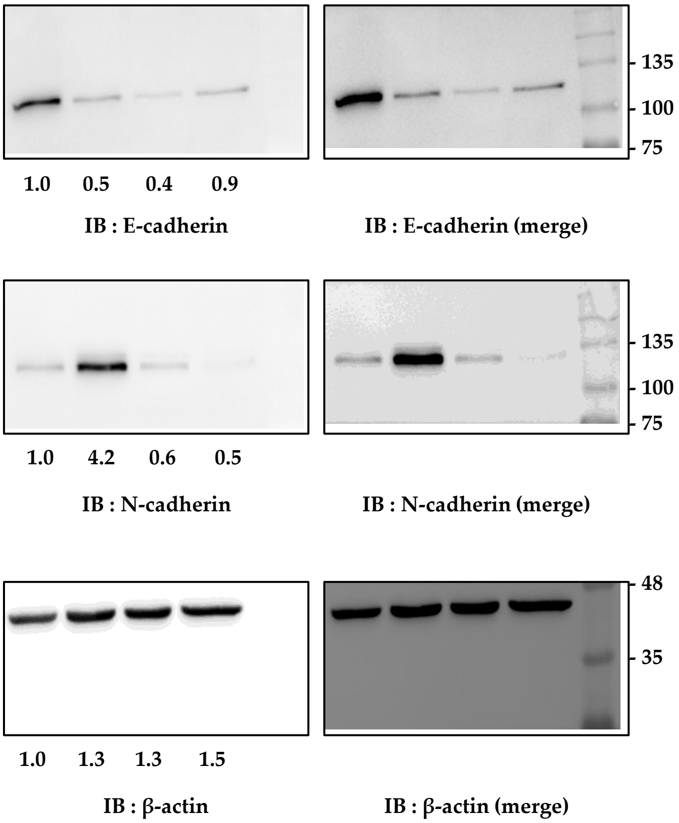
135
100
75
1.0 0.5 0.4 0.9
IB : E-cadherin
IB : E-cadherin (merge)
135
100
75
1.0 4.2 0.6 0.5
IB : N-cadherin
IB : N-cadherin (merge)
48
35
1.0 1.3 1.3 1.5
IB : β-actin
IB : β-actin (merge)

Figure 4A

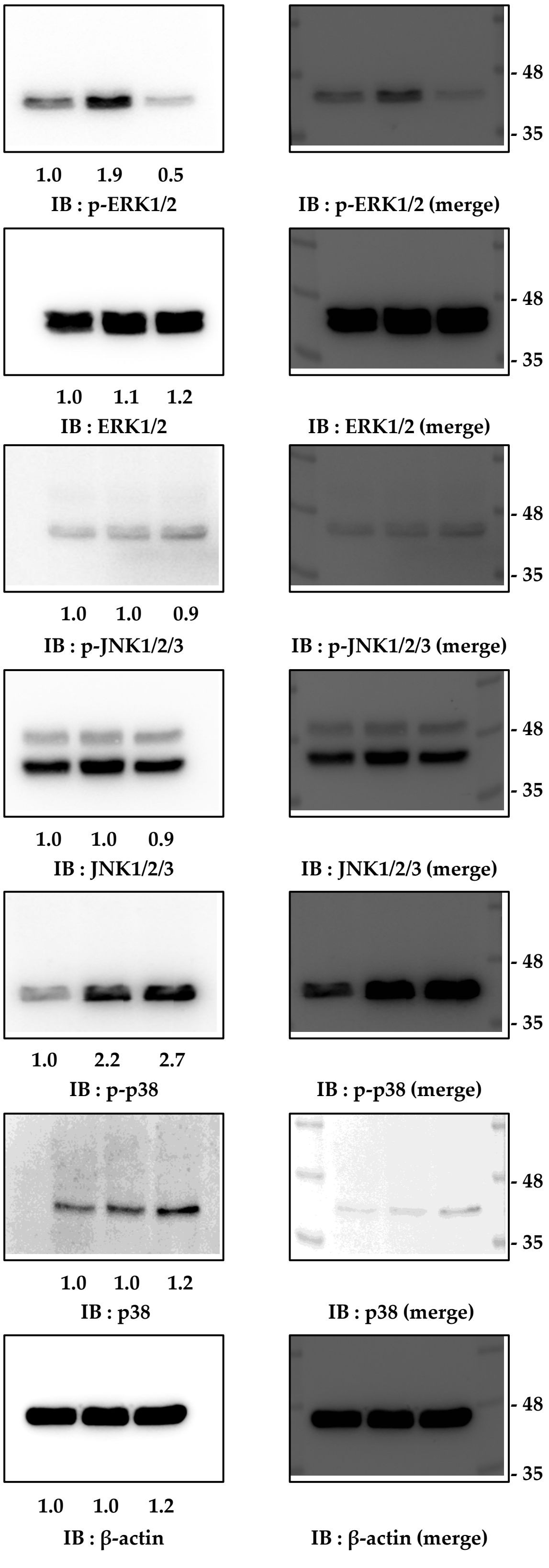

1.0 1.9 0.5
IB : p-ERK1/2
IB : p-ERK1/2 (merge)
- 48
- 35
1.0 1.1 1.2
IB : ERK1/2
IB : ERK1/2 (merge)
- 48
- 35
1.0 1.0 0.9
IB : p-JNK1/2/3
IB : p-JNK1/2/3 (merge)
- 48
- 35
1.0 1.0 0.9
IB : JNK1/2/3
IB : JNK1/2/3 (merge)
- 48
- 35
1.0 2.2 2.7
IB : p-p38
IB : p-p38 (merge)
- 48
- 35
1.0 1.0 1.2
IB : p38
IB : p38 (merge)
- 48
- 35
1.0 1.0 1.2
IB : β-actin
IB : β-actin (merge)
- 48
- 35

Figure 5B

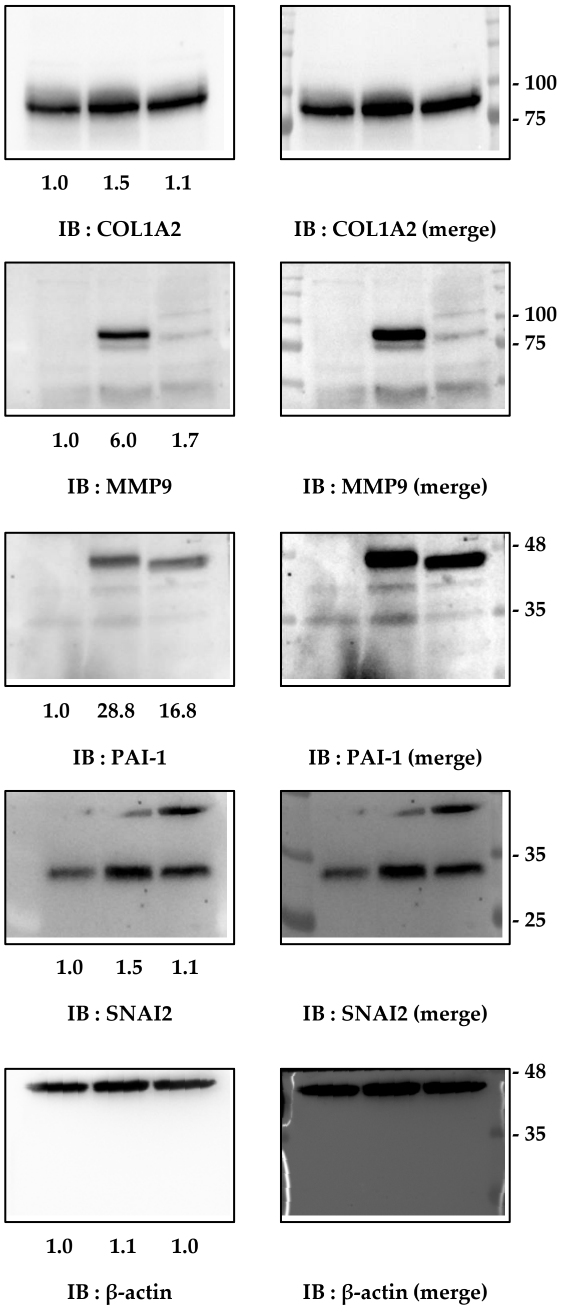

- 100
- 75
1.0 1.5 1.1
IB : COL1A2
IB : COL1A2 (merge)
- 100
- 75
1.0 6.0 1.7
IB : MMP9
IB : MMP9 (merge)
- 48
- 35
1.0 28.8 16.8
IB : PAI-1
IB : PAI-1 (merge)
- 35
- 25
1.0 1.5 1.1
IB : SNAI2
IB : SNAI2 (merge)
- 48
- 35
1.0 1.1 1.0
IB : β-actin
IB : β-actin (merge)
